# The Experiences of Family Caregivers Whose Relative Is Relocating From a Regular Nursing Home to an Innovative Living Arrangement: A Qualitative Study

**DOI:** 10.1111/scs.70257

**Published:** 2026-05-01

**Authors:** Mara Brouwers, Audrey Beaulen, Hilde Verbeek, Wim G. Groen, Bram de Boer, the RELOCARE Consortium, Hilde Verbeek, Jan P. H. Hamers, Jos M. G. A. Schols, Bram de Boer, Judith H. J. Urlings, Mara Brouwers, Wilco P. Achterberg, Monique A. A. Caljouw, Charlotte Poot, Elleke G. M. Landeweer, Dika H. J. Luijendijk, Miranda C. Schreuder, Sytse U. Zuidema, Marieke Perry, Raymond T. C. M. Koopmans, Katrien G. Luijkx, Annerieke Stoop, Wim G. Groen

**Affiliations:** ^1^ Department of Health Services Research, CAPHRI Care and Public Health Research Institute Maastricht University Maastricht the Netherlands; ^2^ Living Lab in Ageing and Long‐Term Care Maastricht the Netherlands; ^3^ Department of Medicine for Older People Amsterdam UMC, Vrije Universiteit Amsterdam Amsterdam the Netherlands; ^4^ Aging & Later Life Amsterdam Public Health Research Institute Amsterdam the Netherlands; ^5^ Ageing & Vitality, Rehabilitation & Development Amsterdam Movement Sciences Amsterdam the Netherlands

**Keywords:** care environment, culture change, dementia, informal caregiver, innovation, innovative housing with care, long‐term care, move, nursing, relocations

## Abstract

**Introduction:**

Due to an ongoing culture change, relocations from regular nursing homes to innovative living arrangements are increasing. This affects not only residents but also their family caregivers. The perspective of family caregivers regarding the relocation processes that occur within the context of culture change is understudied. Therefore, this study examines the experiences of family caregivers concerning the relocation of their relatives from a regular nursing home to an innovative living arrangement.

**Methods:**

A qualitative study, using semi‐structured interviews, was performed in the Netherlands. Three nursing homes offering 24‐h care that were planning a relocation from a regular nursing home to an innovative living arrangement were selected. Twenty‐two family caregivers were included in the study. Interviews took place 2 weeks to a month after the relocation day.

**Results:**

Four themes were constructed from the data: (1) Family caregivers took on an active role throughout the relocation process; (2) Family caregivers were concerned about the emotional impact of relocating on their relative; (3) Lack of communication with family caregivers during the relocation process; and (4) The challenges of implementing the culture change.

**Discussion:**

This study emphasizes the need to provide practical support when preparing for the move. Emotional support, information provision and optimal communication are important to support family caregivers. Furthermore, family caregivers appear to be hesitant regarding the intended culture change. Therefore, it is important to create a sense of innovation readiness in both staff and family caregivers by informing and involving them.

## 1 Introduction

Family caregivers play an important role in caring for their relative, both at home and in a nursing home [1]. They often start caring for their relative long before the actual move to a nursing home, leading to the development of skills and knowledge concerning caregiving [2]. After relocation to a nursing home, staff members aim to keep family caregivers involved, as the importance of their role further increases as their relative's dementia progresses [2, 3]. They can, for example, assure that individual needs of their relative are met and monitor the quality of care [4, 5]. This is especially important during stressful times, such as during a relocation process.

Relocating within long‐term care is impactful and affects not only the residents but also their family caregivers. Most research on this topic focuses on relocations from home to a nursing home [6, 7]. These studies show that family caregivers often report emotional concerns such as grief, stress and shame. Furthermore, they express the need to receive sufficient information and support [8]. However, there is also an increase in the amount of relocations between nursing homes, called mass inter‐institutional relocations. In these types of relocations, all residents and staff move together from one nursing home to another [9]. Research focusing on relocations between nursing homes shows that family caregivers often describe the process of such relocations as chaotic [10]. They worry about how their relative will respond to relocating and do not always feel properly involved [11].

The amount of relocations is increasing due to an ongoing global culture change. This culture change aims to improve residents' quality of life and care in nursing homes by focusing on person‐centred care and de‐institutionalizing nursing homes [12, 13]. This is often achieved by, for example, stimulating autonomy and shared decision‐making, and creating a small‐scale homelike setting [13]. In order to support this culture change, new living arrangements offering 24‐h care are being developed, so‐called innovative living arrangements. These arrangements aim to alter the physical, social and organizational environment. The physical environment includes everything from interior design to the incorporation of nature. The social environment entails how residents interact with others in the environment, and the organizational environment entails how care is organized and what culture an organization adopts [14]. There is a global trend where more alternatives are being developed that radically change these environments compared to regular nursing homes. How these alternatives operationalize is diverse, ranging from an emphasis on creating a community‐like environment to an emphasis on incorporating nature [15, 16, 17].

In the Netherlands, for example, there is a large focus on small‐scale living (e.g., where older adults form a household together with staff members in a small‐scale and homelike environment), green care farms (e.g., where agricultural activities are being combined with care services), dementia villages (e.g., where a local community is created for people with dementia) and intergenerational living (e.g., where students and older adults live together and help each other based on social reciprocity and feelings of community). In other countries, living arrangements with similar values are being developed (e.g., creating a homelike feeling, supporting autonomy, involving family and community, incorporating nature), such as the Green House model in the United States (e.g., a homelike, nature‐based environment with significantly transformed staff roles), the group homes in Japan (e.g., group‐living facilities with an emphasis on familiar relationships and the residents' lifestyle) and shared housing in Germany, where people live together in a small‐scale, homelike environment where family participates in daily life and acts as representative [18]. The development of innovative living arrangements is often regarded as positive, as studies have shown beneficial outcomes in daily life for residents and family [12, 16, 19, 20, 21, 22]. These developments, however, do lead to an increase in relocations.

It is not known how family caregivers experience the relocation of their relatives from a regular nursing home to a newly built innovative living arrangement. As this type of relocation (e.g., a mass inter‐institutional relocation) is occurring more frequently due to the ongoing culture change within long‐term nursing home care and the accompanying development of innovative living arrangements, more insight is needed into these relocations and their impact on the people involved [23]. Although family caregivers play a central role in dementia care and although this role can intensify or change during a culture change [18], their perspective with regard to their experience of the relocation processes that occur within this context of culture change is understudied. Therefore, the research question of this study is: how do family caregivers experience the relocation of their relatives from a regular nursing home to an innovative living arrangement?

## 2 Methods

### 2.1 Study Design

A qualitative study was conducted based on an interpretive description approach, using semi‐structured interviews. This approach was chosen, as this study aimed to capture the subjective experience of family caregivers [24, 25]. In order to ensure transparency and rigour, the consolidated criteria for reporting qualitative research (COREQ) checklist was used for reporting.

### 2.2 Setting and Participants

Nursing homes in the Netherlands that planned a mass inter‐institutional relocation from a regular nursing home to an innovative living arrangement were chosen for this study. These facilities all provided 24‐h care for older adults with dementia. A location was considered to be innovative if it positioned itself as an alternative to a regular nursing home and attempted to change the physical, social and/or organizational environment substantially. In total, three Dutch locations were selected. Family caregivers were included when (1) they had a relative that lived at one of the selected locations and received 24‐h care there; and (2) their relative relocated from the old to the new location.

A maximum variation sampling method was used in this study. A group of family caregivers with a variety of demographic characteristics, such as gender, age, educational level and relationship with the residents were included [26]. Sample size per ward depended on data saturation, achieved when no new themes were observed in the data [27].

### 2.3 Data Collection

Semi‐structured interviews were conducted from June 2021 to May 2022, occurring 2 weeks post‐relocation. The interview topics, outlined in Additional File 1: Supporting information, were structured around the pre‐, during‐ and post‐move phases to include (1) the weeks preceding relocation; (2) the relocation day itself; (3) the initial weeks post‐relocation; and (4) the new location. After care organizations agreed to participate, family caregivers were approached by the care organizations and received an information letter. When interested, they received an informed consent form and were asked to participate. Interviews were conducted either at the locations where the family caregiver's relative lived, by phone or via Microsoft Teams; they were scheduled at the family caregiver's convenience. The interviews were recorded and a summary was sent afterward for a content check. Furthermore, demographic data were collected—that is, age, gender, educational level, number of years their relative had lived in the location and number of times family caregivers visited per week/month.

### 2.4 Data Analysis

Demographic data were analysed using descriptive statistics. Verbatim transcripts of all interviews were analysed thematically [28]. An inductive coding approach was used, guided by the research question [29, 30]. Initially, interviews were comprehensively reviewed to gain familiarity with the data. Summaries of each interview were then made to capture the essence of the interviews and were further validated through content checks with interviewees. Next, the transcripts were labelled and assigned codes. Qualitative data analysis software MAXQDA was used for this purpose [31]. Codes were subsequently organized into thematic clusters. Continuous comparison of interviews, refinement of codes and construction of themes were conducted throughout the analysis. Two researchers (M.B. and A.B.) coded all interviews, 10% of the data being independently analysed by both and compared to ensure analytical rigour. Findings were then discussed within the research team to maintain quality and validity.

### 2.5 Ethics

This study was approved by the Medical Ethics Committee of Zuyderland (registration number: METCZ20210065). This study has been preregistered at ‘onderzoekmetmensen.nl’.

## 3 Results

### 3.1 Sample Characteristics

A total of 22 interviews were conducted, lasting an average of 43 min (range 19–69 min). See Table 1 for participant characteristics.

**TABLE 1:** Participant characteristics.

Demographic variables (mean, SD or number, percentage)	
Age, mean (SD, range)	61.3 (10.5, 43–82)
Gender, number (%)	
Male	5 (22.7%)
Female	17 (77.3%)
Relationship, number (%)	
Partner	4 (18.2%)
Daughter	13 (59.1%)
Son	3 (13.7%)
Son‐in‐law	1 (4.5%)
Niece	1 (4.5%)
Educational level, number (%)	
Preparatory secondary vocational education school	3 (13.6%)
Senior secondary vocational education and training	8 (36.4%)
Senior general secondary education and university preparatory education	7 (31.8%)
Bachelor's or Master's level	4 (18.2%)
Frequency of visits, number (%)	
Every day	3 (13.6%)
More than once a week	10 (45.5%)
Less than once a week	5 (22.7%)
Once a month	1 (4.5%)
Missing	3 (13.6%)

The results highlight that family caregivers have a multifaceted and emotionally engaged role throughout the relocation process. Next to providing practical support, family caregivers' preserve their relatives' comfort and continuity, while simultaneously navigating their own emotional responses. Family caregivers voiced concern about the impact of relocating on their relative. Difficulties in communicating with staff and the challenges associated with culture change further contributed to caregivers' uncertainty and stress. The results section starts with a description of the relocation procedures and the innovative living arrangements relatives relocated to. Furthermore, family caregivers' experiences could be categorized into four themes: (1) Family caregivers took on an active role throughout the relocation process; (2) Family caregivers were concerned about the emotional impact of relocating on their relative; (3) Lack of communication with family caregivers during the relocation process; and (4) The challenges of implementing the culture change.

### 3.2 Relocation Procedures and the New Locations

To prepare for the relocation process, all locations established project and/or working groups that were responsible for either the entire relocation process or a part of it. These groups consisted of managers, nursing staff and client council members. All groups started preparations more than a year before the actual relocation. Next to prepare for the relocation process, all locations also prepared for the initiation of their innovative living arrangement. A project manager, architect and other external parties were actively involved in creating the physical environment and the vision of the new living arrangement. In some locations, staff members were also actively involved in shaping the innovative living arrangement and how it should function. All locations informed stakeholders (such as staff and family members) what the innovative living arrangement would look like, how it would impact the daily lives of the residents and how it would impact the work lives of staff members. Care organizations informed staff and family by organizing informational meetings, letting them visit the location pre‐relocation, and informing them through a face‐to‐face meeting or a mail, folder or letter. The family members and staff members of all locations were given the choice to either relocate to the new location, or relocate to another location of choice. This means that all family members and staff members consciously chose the innovative living arrangement as the new home for their relative and their new workplace. Location B and C also arranged interactive discussions to discuss the envisioned culture change and location C arranged courses, such as practical training in technology use, cooking and how to implement the new care vision. All locations aimed to substantially change the physical (e.g., how the building and its surroundings was constructed), social (e.g., how staff interacted with and cared for residents) and organizational (e.g., how the care vision and task description of staff were constructed) environment. Furthermore, they all aimed to implement an open‐door policy, meaning that residents with dementia could leave the building if preferred, using smart technology.

Location A started as a large building where residents lived on the second floor. It featured multiple, spacious hallways with the rooms situated alongside it. The residents lived together in one of these hallways in smaller groups; one small group of approximately 10 residents was included in this study. Although residents could go outside, there was no garden or green area close by. They relocated to a dementia‐friendly environment, guided by the motto of adding life to days rather than days to life. This is partly achieved by creating different ‘nooks’ all around the building with specific uses. Such as a music room (e.g., with a piano), a game room or a room to withdraw. The aim was to engage in more meaningful activities with residents, and staff members worked in more diverse teams. Staff was stimulated to adjust care to personal needs, wishes and preferences of residents with a specific focus on sense of purpose. This was a big contrast with the way of working at the old location, where the residents were considered challenging due to the behavioural and psychological symptoms of dementia. This led to a primary focus on providing structure and a low‐stimulus environment. The relocation resulted in hiring more staff, who were informed about the new culture. The new facility is a larger building within the village, featuring a lively ‘living kitchen’ at its forefront and with the aim to give residents a central place in the neighbourhood. Involving the neighbourhood and giving them a role in the lives of the residents was stimulated by inviting neighbours to organize a hobby‐related session with residents (e.g., making music together, painting together).

Location B was a large nursing home with multiple wards, where every ward accommodated around 30 residents and was staffed by a substantial number of employees. They moved to small houses accommodating seven residents each, set in a park‐like environment, designed to provide greater freedom of movement and more personalized care. These houses are intended to mimic a typical home, featuring a combined living room and kitchen, a hallway with seven bedrooms and shared bathrooms. To enhance residents' freedom, domotics such as sensors and GPS are utilized. Staff members aimed to form a household together with residents and engage in day‐to‐day activities with them. Focus was on improving autonomy and providing more freedom. Staff had clear differentiated tasks at the old location (e.g., care‐related tasks, domestic‐related tasks), but these tasks were merged at the new location. This meant all staff members engaged in a diversity of tasks, from care‐related tasks and domestic tasks to recreational tasks with residents.

Location C was a building similar to an apartment complex, comprising five floors, each with a kitchen/living room and with approximately seven residents per floor. They relocated to a care environment inspired by green care farms in the countryside, featuring outdoor walking paths and animals. The focus is on fostering residents' strength and independence and engaging in activities together. This meant that staff were trained to engage residents with dementia in daily life by taking care of the animals together, involving them in household tasks and encouraging them to remain as autonomous as possible. This is in contrast to the old location, where staff engaged in more differentiated tasks and usually provided, for example, care and food without actively involving residents within these processes. The new building is spacious, with multiple hallways lined with bedrooms and includes three combined living rooms and kitchens.

### 3.3 Family Caregivers Took on an Active Role Throughout the Relocation Process

Throughout the relocation process, family members had to arrange many practical matters in order to successfully assist their relative with relocating. In the weeks before the relocation day, family caregivers took on the role of packing their relative's belongings. Staff asked family caregivers to finish packing before the relocation day and made sure that relocation equipment, such as boxes, stickers and tape, was provided. Family caregivers were often allowed to decide which room they preferred for their relative and decorated the room to their and their relative's taste. When deciding on the room's location and decoration, their relatives were often not actively involved by the family caregivers and staff members.

We had quite some choice regarding which room. I really tried to think carefully with my sister about what my mother needed. She should not be too far away from everything; she needs to have people around her, so somewhat close to the living group. They really took that into account, which I greatly appreciate. (Daughter, 52, interview f20_03)

The relocation day was mostly experienced by family caregivers as a positive day. The relocation days of all locations were seen as festive. Even though the day was regarded as positive, family caregivers acknowledged it was still impactful and stressful for their relatives. Therefore, the main objective for family caregivers was to distract their relative and make them feel as comfortable as possible. Family caregivers mentioned several points for improvement, ranging from the fact that it was a very long day, to miscommunication and a feeling that the organization was somewhat messy. Because it was a very long day, family caregivers felt sorry for their relatives that they had to spend a long time in the common area.

Family caregivers often visited their relative during the relocation day, either for the entire day or from the moment on that their relative could go to the new location. Family caregivers were allowed to choose whether they wanted to visit, and although some chose not to, most did walk or travel to the new location with their relative.

…and then, at a certain point, we heard we could go there. Therefore, together with the others that were still present, who are going to live with [relative] in the new house, we walked over there. And yes, that was actually quite enjoyable. (Daughter, 61, interview f02_02)

In the preparatory phase and during the relocation day, family caregivers had a clear practical role, but after the resident was relocated, family caregivers became less involved. Some family caregivers helped their relative get used to their new home by visiting more often and then distracting them by chatting or going for a walk. However, most family caregivers did not recollect having actively helped their relative get used to their new location. Most described that staff members had a more active role in this, comforting and distracting the residents.

### 3.4 Family Caregivers Were Concerned About the Emotional Impact of Relocating on Their Relative

Throughout the relocation process, the emotions of family caregivers were closely intertwined with the emotions of their relative. How their relative experienced the relocation impacted the emotions that family caregivers experienced. They had to manage both their own emotions and the emotions of their relative.

Family caregivers had the feeling that the weeks before the actual relocation had a negative impact on their relative. Their relatives showed clear signs of agitation as they noticed a change in their environment. They heard staff and family talking about the relocation and saw belongings being packed. As family caregivers had already noticed that the preparation phase impacted their relative, they expressed worries about the relocation and the new location. They worried, for example, about the fact that their relative would live with new or other residents and staff members, or because the new location would be smaller or bigger in comparison with the old location. They feared the response of their relative or worried about the new location and whether its culture would fit their relatives' needs.

It is, of course, much more small‐scale, and I think that—well, I was a bit worried about it because, as I said, my aunt is quite a solitary person. And in such a small‐scale setting, you might receive more attention than you would want in this case. (Niece, 47, interview f10_02)

One family caregiver even described having had sleepless nights due to the upcoming relocation, as she feared the response of her relative concerning the relocation.

Most family caregivers acknowledged that the relocation day was an impactful day for their relative. Their relatives were overwhelmed by the change in their daily routine, the busy schedule and the new surroundings. Therefore, they displayed signs of stress, being overwhelmed and being exhausted at the end of the day. Although family caregivers appreciated the festive ambience of the relocation day, it was exhausting for them as well, as they had to comfort and support their relative throughout the day. There were some family caregivers that described their relative as calm when relocating: they seemed to accept the interruption in their normal and daily routine. Their relative did not seem to need time to get used to the new location and seemed to feel at home right away.

And the big difference, I have to say, is because of the new location. That is, when she walked in, it was immediate, and you could see it in her reaction, her posture, her body language—‘it is very beautiful here’. (Daughter, 59, interview f31_03)

Family caregivers experienced the weeks after the relocation as either positive or negative. Some family caregivers expressed relief that their relative seemed to feel at home right away, which made them feel happy for their relative. They felt like their relative received more attention at the new location, was more at ease, and showed signs of happiness about their new surroundings.

The whole body language was like, ‘Yes, you know, this is it.’ (…) I got the impression that it was the same for most people, it was like, ‘Ah, space. Peace’. It all looks beautiful and they do experience that. Let me put it this way: their senses do react to the environment. So whether you're in a beautiful area with lots of greenery and open space, or you're trapped in a concrete box, it makes a difference. (Daughter, 59, interview f31_03)

When their relative felt at home, this made family caregivers more satisfied with the new location and made them feel at ease in letting their relative live there. Family caregivers showed happiness when they felt that their relatives' environment improved—for example, when they felt like their resident had more freedom at the new location, or when they had the feeling that staff caregivers were very empathic to their relative, giving the family a feeling of trust.

I think primarily the fact that it is a beautiful environment, it is peaceful—that has had a particularly positive effect on all of us. And naturally, you want the best possible surroundings for your loved ones (…) with a nice, enthusiastic team. So that is really just wonderful. I think the effect on us has been positive. (Son, 55, interview f07_04)

Furthermore, family caregivers expressed happiness when their relative moved back to their birth village. This because they valued the fact that the surroundings would be familiar for their relative. Furthermore, it enabled their relative to live close to his/her family once again.

Some caregivers, however, felt that the relocation has had a negative impact on their relative. Residents showed signs of not feeling at home, being restless and agitated. They often expressed that they wanted to ‘go home,’ indicating that they did not see their new environment as their home. Family caregivers whose relative did not feel at home were less satisfied with the process and felt that the relocation had negatively affected their relative. This made it more difficult for them to leave their relative behind and made some family caregivers feel sad.

And then he says: ‘Do I have to sleep here?’ Well, yes. It is sad, but there is no other choice. I would rather take him home with me. (Partner, 82, interview f15_03)

### 3.5 Lack of Communication With Family Caregivers During the Relocation Process

In the weeks before the relocation, family caregivers had to inform their relative. As all residents had dementia, family caregivers often chose a distinct strategy to inform their relative. Family caregivers either chose to inform relatives as late as possible to diminish stress, or they repeated information often throughout the weeks, as they hoped this would increase the chance that their relative remembered that they were going to relocate. There was a lot of uncertainty about what strategy would be best for their relative. Family caregivers often expressed not knowing how their relative would react to the upcoming relocation. Staff also had an active role in informing residents about the upcoming relocation, but they did not always adequately communicate their strategy with family caregivers. Family caregivers did not always feel properly involved, which increased their stress. They either did not receive proper information, or they had the feeling that staff members were not involved either. In comparison, family caregivers that felt properly informed experienced less stress.

Furthermore, the strategy that staff members used differed between locations. One location started informing early and used multiple strategies, such as visiting the new location beforehand, having a calendar that counted down to the relocation day, or informally chatting about the location. Another location, however, did not inform the residents at all until it was no longer avoidable. Several family caregivers mentioned that it was unclear for them how their relative was informed by staff, which also made it more difficult for them to determine when and how to discuss the upcoming relocation with their relative.

At one point, it was suggested not to start talking about the move too early. Because, you know, they might forget again, and so on. There was not really any coordination on when we should or shouldn't discuss it. So, that could be something to pay attention to. (Son, 61, interview f05_04)

During the relocation day, some family caregivers felt like the staff members did not really know what to do, leading to missing belongings, or furniture that was not in the right place. Family caregivers were disappointed for their relative that this did not go as smoothly as expected. For themselves, this felt ‘messy’ as well.

I got the impression that it was just chaotic. No one knew what was going on. I kind of felt as if they did not really know where everything was or what was supposed to happen. (…) My impression was that they did not really know what they were doing either. There did not seem to be a plan. That was the impression I got. (Daughter, 59, interview f31_03)

In the weeks after the relocation, family caregivers tried to contact staff members in order to advocate for the best interests of their relative. For all locations, contact with staff became more difficult after the relocation, due to needing to hire more staff, or the decision to mix staff teams and residents into new groups. Family members found it most important that they knew the staff members and knew who to approach. It was seen as positive when the staff members relocated with their relative to the new location. However, due to the hiring of new staff members, this was often difficult. For family caregivers, all these new faces were challenging. They had to get used to the new staff members and they were unsure who to approach.

There are some new health care staff now, and well, everything should be properly outlined in a care plan in theory, but that handover is still sometimes, well, sometimes you still need to point their attention to certain matters. (Daughter, 56, interview f03_01)

Family caregivers either experienced that staff members were harder to find due to the small‐scale nature of the new location (e.g., fewer staff members per ward), or they felt contact became less personal due to an increase in the number of staff members. They lacked clarification about who would be their point of contact and often struggled to find staff members who could answer their questions.

### 3.6 The Challenges of Implementing the Culture Change

Family caregivers were very satisfied with the physical environment. Their overall view was that the new location was very beautiful. They felt their relatives' conditions improved when compared to the old location. Family caregivers especially appreciated the facilities, such as having a restaurant that is closer by and having facilities that are neat and clean. They felt that their relative profited from these facilities and it made visiting their relative much more pleasant. They especially appreciated the fact that the new location was more spacious and had better lighting. Furthermore, there was a larger outside area at all the new locations. This was highly appreciated by family caregivers, as they felt that their relative was better able to go outside and enjoy the beautiful surroundings and nature. The old locations did not have a lot of opportunities to enjoy nature, as they did not have extensive outside areas and nature was challenging to reach due to distance. They were happy about the ability to take their relative for a walk when they visited.

It is wonderful for my father that he can go outside. If the weather is nice, we can go outdoors and I can take a walk with him. That means we do a lot of uphill and downhill; I have to push the wheelchair quite a bit. Yes, it's—it's an unique experience in terms of location. (Daughter, 62, interview f07_03)

Family caregivers also mentioned that the ambience was improved at the new location. It felt more cozy and homely, and it was nice to sit in the communal areas or at the restaurant.

Although family caregivers showed a lot of enthusiasm regarding the physical environment, they were more critical about the culture change or ‘new way of working’ at the new location. Family caregivers either did not agree with the new culture or experienced a lot of uncertainty as to how the culture change was intended. Furthermore, they doubted whether the culture change was successfully implemented as intended, or they did not notice a difference in the way of working. Family caregivers struggled with some aspects of the culture change. One location intended to open the location in order to give residents more freedom. However, this made family caregivers feel uneasy, as they were afraid this would not be achievable in practice.

I already had my reservations about it, because they start walking and suddenly end up somewhere without knowing where they are. They do not have enough staff to keep track of all these movements and assess whether or not they need to intervene. That's the thing with this system that tracks someone's location—you can do it with a phone, but you cannot assess from a distance whether you need to step in. (Daughter, 59, interview f31_03)

The new locations depended more on technology, which did not always work as intended, making the family caregivers feel uneasy. For example, in one location, there was no longer a standard night shift. Instead, the staff depended on monitoring at a distance using sensors and cameras, which led to insecurity in both family caregivers and staff. One family caregiver mentioned that their relative had fallen during the night and was not discovered for quite some time, which made the family caregiver feel distressed. Staff also depended on sensors to be able to increase the freedom of movement for residents. They would receive a sign when residents went outside or family caregivers wanted to enter, but these sensors did not always work as intended.

Furthermore, family caregivers felt that staff members struggled with implementing the culture change. They noticed in the behaviour of staff that they were not yet comfortable with the new way of working and struggled to implement this way of working in everyday life. Furthermore, as technology was not yet reliable enough and there was some insecurity about the implementation of the culture change, some goals were not yet pursued, such as the intended open‐door policy. Family caregivers felt that this led to dissatisfaction among staff members.

I think there's something not quite right within the organization or in the way—yes, that they had a different expectation of how the work would be. Maybe it was presented to them differently as well. I am not sure. But there is some dissatisfaction there, and that naturally affects the care being provided, right? (Partner, 75, interview f13_04)

## 4 Discussion

This study explored the experiences of family caregivers whose relative relocated from a regular nursing home to an innovative living arrangement. Family caregivers played an important and large role throughout the relocation process. These roles were practical (e.g., packing belongings, personalizing new room) and emotional (e.g., distracting their relative, comforting them). Family caregivers worried about the impact of the relocation on their relative and displayed emotional distress when their relative did not seem to settle in. Family also had to communicate with their relative and staff members throughout the relocation process, which was perceived as challenging. It was difficult to determine the right moment to inform their relative about the upcoming relocation. Furthermore, after the relocation, communication with staff became more challenging due to changes in staffing and changing roles of staff members. Finally, family caregivers assessed the new environment after the relocation. Although family caregivers mostly described the physical environment as beautiful, they were more critical concerning the culture change that took place.

The results show that family caregivers whose relative relocates towards an innovative living arrangement encounter similar challenges as family caregivers whose relative relocated between regular nursing homes. Family caregivers experience feelings of stress, they feel as if the relocation process is chaotic, and they do not always feel properly involved. This is in line with previous studies looking into relocations between regular nursing homes [10, 11]. However, this study shows that relocating to an innovative living arrangement poses additional challenges, but also additional opportunities. One opportunity, for example, is the fact that family members are very positive concerning the new environment, which is barely mentioned in studies focusing on relocations between regular nursing homes. This positive attitude regarding the new environment gives opportunity to create a new home for their relatives, where family caregivers can take in an active role. The buildings are consciously constructed to create a homelike environment and ambiance, which is crucial for feeling at home and creates the opportunity for family caregivers to become part of the household [13, 32]. The recognizable environment creates more opportunity to develop meaningful relationships with family and other familiars [33, 34]. The outside area is designed to attract residents and serve individual and collective needs, which also offers opportunities for family caregivers to engage in more meaningful activities with their relative (e.g., feeding animals together) [35, 36]. This is in line with literature, showing that innovative living arrangements often offer more opportunities for family members to be actively involved in the daily lives of residents and form a partnership with staff [20, 37]. Furthermore, family caregivers of residents living in a small‐scale environment, which is a type of innovative living arrangement, were more positive in general compared to those whose residents lived in regular nursing home settings [19, 20]. This however, requires more than an improved physical environment alone. In order to create an effective family–staff relationship, staff members need a shift in mindset and regard the family caregiver as full care partner. This emphasizes the need for staff members to actively share information, involve family caregivers in care and share responsibility [38]. The results of our study shows that family caregivers do not yet feel optimally involved and informed, meaning that the locations we followed did not yet succeed in creating an optimal family–staff relationship. A relationship that is crucial, as research shows that active participation of family members leads to an improved quality of life of residents in terms of social relationship and social isolation [37].

Relocating to an innovative living arrangement also poses challenges. The results indicate that family caregivers have a critical attitude concerning the intended culture change. They seem to be hesitant about the new culture, on aspects such as new technology, new staff roles and the open‐door policy. Although most family caregivers saw a lot of potential in, for example, opening the doors for their relative, they also voiced a lot of concern whether staff would be able to guarantee the residents' safety. An important aspect to take into account when implementing a new culture is the change readiness of all stakeholders involved. Change readiness is the belief that changes are needed, which leads to accompanying attitudes and intentions necessary to successfully implement these changes [39]. Although most family members acknowledged the need for change, our results show that family members were critical whether the intended culture change provided this needed change. Most family caregivers acknowledged that a change was needed. However, a successful change also requires a belief that a change will have positive outcomes, and that the group has the capability to successfully undertake change [40]. Family caregivers were not convinced that the envisioned changes would lead to positive outcomes. For example, family members did not consider the use of a GPS tracker as an optimal alternative to provide residents with more freedom. Furthermore, family members noticed that staff members seemed to struggle with the new way of working, which in turn raised doubt whether the current group of staff members had the capability and required competencies to successfully undertake this change. However, the interviews were conducted 2 weeks after the relocation day. Previous studies show that culture change is a long pending and intensive undertaking and staff often experience several barriers that complicate a successful culture change [13, 41, 42]. Having room for learning is essential in creating a climate that stimulates innovation.

### 4.1 Methodological Considerations

Research shows that culture change is a long and complicated process, meaning that the culture change is not completely, or even partly, implemented until some time after the relocation. As we wanted to avoid recall bias, we planned the interviews with family caregivers 2 weeks after the actual relocation day. Future follow‐up studies could be performed to gain more understanding into the culture change and how this develops further after a relocation. Furthermore, most relocations took place during COVID. This means that not all preparations could proceed as planned. This could also have affected the communication between family caregivers and staff members, as rules concerning visiting hours and staff's way of working were stricter.

### 4.2 Implications for Research and Practice

This study shows that a relocation to an innovative living arrangement is an impactful undertaking. In the first place for residents, but also for staff and family caregivers. This study shows that family caregivers play a large role throughout a relocation process and should be supported and facilitated in order to optimize such an undertaking, making sure that residents feel at ease during the process. The relocation process itself is impactful and family caregivers expressed several barriers that should be taken into account. In the weeks before the actual relocation day, the emphasis should be on providing practical support for preparing for the move and emotional support, as relocating is seen as a stressful undertaking. The provision of detailed information is an important aspect that made family members feel more at ease. After the relocation day, it is important to optimize communication between the family caregiver and staff members further.

Moving to an innovative living arrangement also poses additional challenges. This study shows that family caregivers appear to be hesitant regarding the intended culture change. Therefore, it is important to create a sense of innovation readiness in both staff and family caregivers by informing and involving them. Follow‐up research should take these additional challenges into account. More research into how to prepare residents and family caregivers for a new culture should be conducted. Furthermore, more insight into how to create a sense of innovation readiness and how to optimally involve family caregivers in the culture change process is needed. The results show that family caregivers have an important role to play in supporting their loved ones throughout a relocation process. Therefore, giving them the tools and support to do so is crucial.

## Author Contributions

M.B. and A.B. conducted the analysis. M.B. wrote the manuscript, and A.B., H.V, W.G.G. and B.B. revised the manuscript. All authors have critically read and approved the final manuscript.

## Funding

This work was supported by the RELOCARE consortium who received a grant from the Dutch Ministry of Health, Welfare, and Sport (grant no. 330436).

## Conflicts of Interest

The authors declare no conflicts of interest.

## Supporting information


**Data S1:** Ervaring omtrent verhuizing: Naaste.

## Data Availability

The data that support the findings of this study are available on reasonable request from the corresponding author.
