# Supplementary material for: The Experiences of Family Caregivers Whose Relative Is Relocating From a Regular Nursing Home to an Innovative Living Arrangement: A Qualitative Study
Source: Scand J Caring Sci. 2026 May 1;40:e70257. doi: 10.1111/scs.70257 (PMC13135105; doi:10.1111/scs.70257)
Supplement: Supplementary file 1 — Data S1: Ervaring omtrent verhuizing: Naaste. [file SCS-40-0-s001.docx]

Ervaring omtrent verhuizing: Naaste

| **Ervaringen van naaste rondom de verhuizing** | | |
| --- | --- | --- |
| **Inleiding interview:** Ik wil het graag met u hebben over de verhuizing die heeft plaatsgevonden. Ik ben vooral geïnteresseerd in hoe u deze verhuizing heeft ervaren en wat u als positief en negatief hebt ervaren tijdens het verhuizen. Hier zullen de meeste vragen dan ook over gaan. Graag zou ik willen beginnen met een brede algemene vraag: Hoe bevalt de nieuwe locatie? | | |
| **Hoofdvraag** | **Interviewvragen** | **Categorie** |
| **Hoe heeft de naaste van de bewoner het verhuizen van of naar een innovatief woonzorgconcept ervaren?**  **Welke aspecten heeft hij/zij als negatief ervaren en welke als positief?** | Hoe bevalt de nieuwe locatie? | Initiële vraag |
|  | Hoe is de aanloop naar de verhuizing verlopen?   - Op welke wijze heeft u zich voor kunnen bereiden op de verhuizing? - Op welke wijze werd uw naaste voorbereid en geïnformeerd? - Wanneer werd u geïnformeerd over de verhuizing? - Op welke wijze werd u geïnformeerd over de verhuizing? (Bv. Zijn er meetings georganiseerd et cetera) - Heeft u de mogelijkheid gehad de nieuwe locatie te bezoeken? Zo ja, hoe heeft u dit bezoek ervaren? - Hoe heeft u de weken voor het verhuizen ervaren? | Aanloop verhuizing |
|  | Hoe is de verhuizing zelf verlopen?   - Zou u me stap voor stap uit kunnen leggen hoe de verhuizing is verlopen? (Bijvoorbeeld, wat was de gemoedstoestand van uw naaste, werd u geholpen voor personeel) - Vond u de verhuizing goed georganiseerd? Zo ja, waarom wel? Zo nee, waarom niet? - Hoe heeft u de verhuizing van uw naaste ervaren? | Verhuizing |
|  | Hoe is het gewennen van uw naaste aan het nieuwe gebouw verlopen?   - Hoe was de gemoedstoestand van uw naaste gedurende de eerste week? - Hoe heeft u de eerste week na de verhuizing ervaren? - Op welke wijze werd uw naaste geholpen met het gewennen aan de nieuwe omgeving? | Nasleep verhuizing |
|  | Hoe bevalt u de nieuwe omgeving?   - Hoe bevalt de nieuwe indeling van de gebouwen? - Hoe bevalt de nieuwe werkwijze van het personeel? - Hoe bevallen de nieuwe faciliteiten? | Nieuwe locatie |
